# Supplementary material for: Structure and topology around the cleavage site regulate post-translational cleavage of the HIV-1 gp160 signal peptide
Source: eLife. 2017 Jul 28;6:e26067. doi: 10.7554/eLife.26067 (PMC5577925; doi:10.7554/eLife.26067)
Supplement: Figure 4—source data 1. — Summary data of FRAP analysis. Experiments were performed as described in Figure 4C. n: number of cells analyzed per experiment. [file elife-26067-fig4-data1.docx]

| **Reporter** | ***D* (µm/s^2^) ± SD** | **n** |
| --- | --- | --- |
| ER-GFP | 10.2 ± 1.6 | 13 |
| SP+1 | 9.6 ± 2.7 | 11 |
| SP+10 | 1.7 ± 0.6 | 11 |

**Figure 4 – Source data 1: Summary of FRAP data**

Summary data of FRAP analysis. Experiments were performed as described in Figure 4C. n: number of cells analyzed per experiment.
