# Supplementary material for: Structure and topology around the cleavage site regulate post-translational cleavage of the HIV-1 gp160 signal peptide
Source: eLife. 2017 Jul 28;6:e26067. doi: 10.7554/eLife.26067 (PMC5577925; doi:10.7554/eLife.26067)
Supplement: Figure 7—source data 1. — Graphpad was used to calculate all statistical values for the above time points. p values were calculated using an unpaired, two-tailed t test with Welch’s correction. [file elife-26067-fig7-data1.docx]

| Antibody | Chase | % recognition  (mean) | | SD | | n | p |
| --- | --- | --- | --- | --- | --- | --- | --- |
| D7324 |  | Wt | M26P | Wt | M26P |  |  |
|  | 0’ | 105 | 84 | 65 | 45 | 3 | 0.669 |
|  | 30’ | 91 | 72 | 26 | 3 | 3 | 0.277 |
|  | 2h | 94 | 74 | 6 | 2 | 3 | 0.00541 |
| A32 |  |  |  |  |  |  |  |
|  | 0’ | 7 | 2 | 5 | 2 | 3 | 0.183 |
|  | 30’ | 13 | 4 | 5 | 3 | 3 | 0.0556 |
|  | 2h | 18 | 8 | 3 | 4 | 3 | 0.0257 |
| CD4-IgG |  |  |  |  |  |  |  |
|  | 0’ | 7 | 7 | 1 | 3 | 3 | 1.0 |
|  | 30’ | 43 | 27 | 9 | 5 | 3 | 0.0546 |
|  | 2h | 65 | 54 | 21 | 6 | 3 | 0.432 |

**Figure 7 – Source data 1:**

Autoradiographs from Figure 7F were quantified and each antibody normalized to the recognition of polyclonal antibody 40336 which recognizes all forms of gp160. Graphpad was used to calculate all statistical values for the above time points. p values were calculated using an unpaired, two-tailed t test with Welch’s correction.
